# Effects of Dapagliflozin on Body Composition and Liver Tests in Patients with Nonalcoholic Steatohepatitis Associated with Type 2 Diabetes Mellitus: A Prospective, Open-label, Uncontrolled Study

**DOI:** 10.1016/j.curtheres.2017.07.002

**Published:** 2017-07-08

**Authors:** Hiroshi Tobita, Shuichi Sato, Tatsuya Miyake, Shunji Ishihara, Yoshikazu Kinoshita

**Affiliations:** Department of Gastroenterology and Hepatology, Shimane University Faculty of Medicine, Izumo, Japan

**Keywords:** nonalcoholic, steatohepatitis, type 2 diabetes mellitus, sodium–glucose, cotransporter 2-inhibitor, dapagliflozin, open-label

## Abstract

**Background:**

Nonalcoholic steatohepatitis (NASH) is an active form of nonalcoholic fatty liver disease. Risk factors for NASH include type 2 diabetes mellitus (T2DM) and obesity. Sodium–glucose cotransporter 2 (SGLT2) inhibitors used to treat T2DM prevent glucose reabsorption in the kidney and increase glucose urinary excretion. Dapagliflozin is a potent, selective SGLT2 inhibitor that reduces hyperglycemia in patients with T2DM and has been demonstrated to reduce some complications associated with NASH in rodent models.

**Objective:**

To assess the efficacy and safety profile of dapagliflozin for the treatment of NASH-associated with T2DM.

**Methods:**

In this single-arm, nonrandomized, open-label study, 16 patients with percutaneous liver biopsy-confirmed NASH and T2DM were enrolled to be prescribed dapagliflozin 5 mg/d for 24 weeks. Of these, 11 patients were evaluable. Patients with chronic liver disease other than NASH were excluded. Body composition, laboratory variables related to liver tests and metabolism, and glucose homeostasis were assessed at baseline and periodically during the study. Changes from baseline were evaluated with the Wilcoxon signed-rank test.

**Results:**

Administration of dapagliflozin for 24 weeks was associated with significant decreases in body mass index (*P* < 0.01), waist circumference (*P* < 0.01), and waist-to-hip ratio (*P* < 0.01). Changes in body composition were driven by reductions in body fat mass (*P* < 0.01) and percent body fat (*P* < 0.01), without changes in lean mass or total body water. Liver tests (ie, serum concentrations of aspartate aminotransferase, alanine aminotransferase, ferritin, and type IV collagen 7S) also significantly improved during the study. Insulin concentrations decreased (*P* < 0.01 by Week 24) in combination with significant reductions in fasting plasma glucose (*P* < 0.01) and glycated hemoglobin (*P* < 0.01) levels and increases in adiponectin (*P* < 0.01) levels from Week 4 onward.

**Conclusions:**

Dapagliflozin was associated with improvements in body composition, most likely a reduction in visceral fat, which occurred together with improvements in liver tests and metabolic variables in patients with NASH-associated with T2DM.

UMIN Clinical Trial Registry identifier: UMIN000023574.

## Introduction

Nonalcoholic steatohepatitis (NASH) is an active form of non-alcoholic fatty liver disease (NAFLD). NASH is characterized by steatosis, liver fibrosis, ballooned hepatocytes, and lobular inflammation. In some patients, NASH may progress to cirrhosis of the liver and hepatocellular carcinoma.^1^ NAFLD significantly increases the risk of incident type 2 diabetes mellitus (T2DM) and metabolic syndrome.^2^ Significant risk factors for NASH are T2DM and obesity.^3^, ^4^

The preferred therapeutic approach is to encourage lifestyle changes to reduce a patient’s weight by ≥7% through changes in diet and lifestyle habits, including regular exercise.^5^ However, a pharmacologic approach is often necessary to treat NASH because most patients fail to reduce their weight sufficiently. Pharmacologic options include pioglitazone and metformin, both of which are insulin-sensitizing agents^6^ that are used to treat T2DM.^7^, ^8^ Studies of these drugs have reported their effectiveness in the treatment of NASH and its comorbidities.^7^, ^8^ However, pioglitazone may promote weight gain, is not available in some countries, and should not be used in patients with heart failure. A network meta-analysis found some evidence for improvements in histologic features of NASH with thiazolidinediones.^9^ However, a recent Cochrane review reported uncertainty about the effects of pharmacotherapy on NAFLD and hepatic steatosis owing to low-quality evidence.^10^ In general, alternative treatments of NASH associated with T2DM are warranted.

Sodium–glucose co-transporter 2 (SGLT2) inhibitors prevent the reabsorption of glucose in the kidney and increase urinary excretion of glucose,^11^ and several members of this class have been approved for the treatment of T2DM in human beings. Recent studies revealed that SGLT2 inhibitors have therapeutic effects on NASH in rodent models,^12^, ^13^, ^14^ raising the possibility that they may be beneficial in treating NASH associated with T2DM. To date, no studies have evaluated the effects of SGLT2 inhibitors for the treatment of NASH associated with T2DM in human beings.

Dapagliflozin is a potent and selective SGLT2 inhibitor that has been shown to reduce hyperglycemia in patients with T2DM.^15^ Dapagliflozin was also reported to reduce some of the complications associated with NASH in rodent models.^16^, ^17^ Therefore, in the present study, we evaluated the effects of dapagliflozin for the treatment of NASH in patients with T2DM. Eligible patients were administered dapagliflozin for 24 weeks, and its therapeutic effects were evaluated by measuring serum biochemistry parameters and performing body composition tests.

## Patients and Methods

### Study design

The study was a prospective, open-label, uncontrolled pilot study. No formal sample size calculation was undertaken; however, the number of patients was estimated to be 20.

### Ethics

This clinical study was performed after obtaining approval from the Ethics Committee of Shimane University School of Medicine, as well as written informed consent from all participating patients. This study complied with the Declaration of Helsinki and applicable laws and requirements. The trial was registered at the University Hospital Medical Information Network under the registration number UMIN000023574. All costs were covered by patients because the study was carried out as part of standard care in daily clinical practice under Japanese health insurance. This was an investigator-initiated study; the sponsor only provided funding for writing support.

### Patients and administration of dapagliflozin

Patients were screened for metabolic syndrome, T2DM, NASH, dyslipidemia, and hypertension, with all current medications recorded. Metabolic syndrome was defined as described previously, with minor modifications.^18^, ^19^ Specifically, participants having at least 3 of the following 5 clinical measures were considered to have metabolic syndrome: central obesity (waist circumference ≥90 cm in men or ≥85 cm in women); elevated blood pressure, defined as systolic blood pressure ≥130 mm Hg or diastolic blood pressure ≥85 mm Hg, or taking an antihypertension medication; elevated fasting blood glucose level ≥110 mg/dL or taking a hypoglycemia medication; decreased HDL-C level <40 mg/dL; and hypertriglyceridemia (≥150 mg/dL) or taking a lipid-lowering medication. T2DM was diagnosed as fasting plasma glucose (FPG) ≥126 mg/dL and glycated hemoglobin (HbA1c) ≥6.5%.^20^ A percutaneous liver biopsy under ultrasound guidance was carried out for patients who had alcohol consumption lower than 20 g/d and who were negative for Wilson’s disease, hemochromatosis, hepatitis B, and hepatitis C, as well as being negative for the presence of antinuclear antibodies and antimitochondrial antibodies. Based on standard clinicopathologic criteria,^21^, ^22^ patients were finally diagnosed as having NASH if they had steatosis, hepatocyte ballooning, and lobular inflammation, with or without fibrosis in the biopsy. After the baseline assessment, all patients were prescribed once-daily dapagliflozin at a dose of 5 mg/d for 24 weeks.

### Measurement of body composition

Body composition was measured at baseline and at Weeks 2, 4, 8, 12, 16, 20, and 24 by segmental multifrequency bioimpedance analysis with an InBody720 (Biospace, Denver, Colorado).^23^ Waist circumference was measured at the middistance between the bottom of the rib cage and the top of the iliac crest using inelastic tape.

### Serum biochemistry

We analyzed the following serum liver tests and metabolic variables: aspartate transaminase (AST), alanine transaminase (ALT), FPG, insulin, HbA1c, HDL-C, LDL-C, and triglycerides. In addition, the following laboratory tests were performed: type IV collagen 7S (T4C7S), ferritin, and adiponectin, as well as the NAFIC score and Fibrosis-4 index, γ-glutamyl transpeptidase, and high sensitivity C-reactive protein. The NAFIC score is useful for predicting steatohepatitis in nonalcoholic fatty liver disease and is calculated from the levels of ferritin, fasting insulin, and T4C7S.^24^, ^25^ The Fibrosis-4 index is calculated as: (age [years] × AST [U/L]) / (platelet count [10^9^/L] × ALT [U/L]).^26^

### Statistical analysis

Data are expressed as the median (interquartile range). The Wilcoxon signed-rank test was used to compare the values obtained at baseline with those obtained at Week 24 in patients who completed the study. *P* < 0.05 was considered to indicate statistical significance of changes versus baseline.

## Results

### Patients

**Figure 1:**
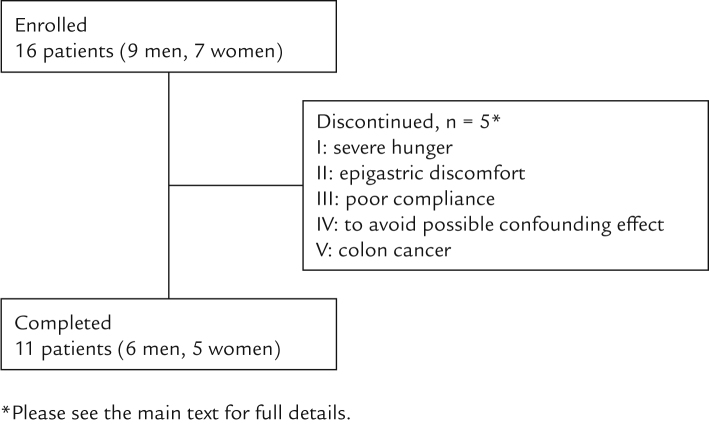
Patient flow and reasons for withdrawal from the study. ^*^See the main text for full details.

Sixteen patients (9 men and 7 women) with a median age of 58 years were enrolled during the study period. Five patients discontinued or were excluded from the final analyses for the following reasons: 1 patient reported severe hunger and another patient reported epigastric discomfort, both after 4 weeks of treatment with dapagliflozin; 1 patient was excluded after Week 12 owing to poor compliance (it was determined during the consultation that the drug was being taken approximately once every 3 days); 1 patient received glimepiride before starting this study and continued administration during the study (the glimepiride dose was reduced from 1 to 0.5 mg at the patient’s request in Week 16, yet this patient was excluded from the study to avoid possible confounding effects of the change in glimepiride dose on laboratory variables); and 1 patient was confirmed to have colon cancer after Week 20. Therefore, 11 patients (6 men and 5 women) aged 46 to 78 years (median, 53 years) completed the study (Figure 1).

The characteristics of these 11 patients are summarized in Table I. All patients had received nutrition and exercise guidance for ≥6 months before enrollment, but these lifestyle interventions were insufficient in terms of reducing the patients’ body weight (producing neither the targeted ≥7% reduction in body weight nor a decrease in ALT value).

**Table I:** Characteristics of individual patients.

Case⁎	Age	Sex	Metabolic syndrome	Hypertension	Dyslipidemia	Concomitant drugs
B	55	M	+	+	+	DPP4i, bezafibrate, ezetimibe
C	78	M	+	+	−	DPP4i, Ca blocker, ARB
E	67	F	+	+	+	DPP4i, αGI, SU, BG, statin
F	76	F	+	+	−	DPP4i, ARB, α-tocopherol
G	50	F	−	−	+	Statin, EPA, DHA, α-tocopherol
H	50	M	+	−	+	α-Tocopherol
I	68	F	−	−	−	DPP4i, EPA
J	47	M	+	+	+	DPP4i, BG, ezetimibe, ARB, α-tocopherol
M	52	F	+	−	+	DPP4i, BG, αGI, ezetimibe
N	46	M	−	+	−	DPP4i, α-tocopherol
P	47	M	+	+	+	DPP4i, Ca blocker

αGI = α-glucosidase inhibitor; ARB = angiotensin II receptor blocker; BG = biguanide; Ca= calcium; DHA = docosahexaenoic acid; DPP4i = dipeptidyl peptidase-4 inhibitor; EPA = eicosapentaenoic acid; SU = sulfonylurea.

⁎ Cases A, D, K, L, and O were excluded for the reasons described in the Results section.

### Concomitant diseases and medications

Metabolic syndrome was diagnosed in 8 patients and hypertension was diagnosed in 7 patients (Table I). Dyslipidemia including abnormal HDL-C, LDL-C, and triglyceride concentrations was diagnosed in 7 patients (Table I).

Of 7 patients diagnosed with hypertension, 4 had been prescribed antihypertension drugs (Table I). Of 7 patients who were diagnosed with dyslipidemia, 5 had been prescribed antidyslipidemia drugs (Table I). In addition, 9 patients were taking a dipeptidyl peptidase-4 (DPP4) inhibitor, 1 patient was taking a sulfonylurea, and 3 patients were taking metformin (Table I). All patients reported that the type and dose of these drugs had not changed for ≥6 months before enrollment.

### Body composition

Table II shows the body composition at baseline and at each visit after starting dapagliflozin. The median baseline body weight was 79.6 kg (interquartile range, 63.3–94.2 kg) and the median body mass index (BMI) was 31.0 (range, 27.0–32.5). BMI exceeded 25 in 9 out of 11 patients. The median waist circumference and waist-to-hip ratio were 101.4 cm (range, 97.6–108.5 cm) and 1.02 (range, 0.96–1.05), respectively. The median body fat mass, skeletal muscle mass, and total body water were 28.3 kg (range, 25.7–35.4 kg), 24.6 kg (range, 21.1–36.3 kg) and 32.7 L (range, 28.8–47.8 L), respectively.

**Table II:** Effects of dapagliflozin on body composition-related variables (n = 11).

	Week 0 (baseline)	Week 2	Week 4	Week 8	Week 12	Week 16	Week 20	Week 24
	Median (interquartile range)							
Body weight (kg)	79.6 (63.3–94.2)	78.3 (62.7–93.2)**	78.5 (62.8–92.8)*	78.8 (61.7–88.3)**	79.7 (60.8–85.3)**	79.6 (59.8–82.9)**	77.5 (60.4–81.7)**	75.8 (59.8–82.3)**
BMI	31.0 (27.0–32.5)	30.3 (26.9–32.2)**	29.9 (27.2–32.1)*	29.0 (26.8–31.7)**	28.1 (26.2–31.7)**	27.4 (25.6–31.8)**	27.6 (25.3–31.3)**	27.3 (24.8–31.3)**
Waist circumference (cm)	101.4 (97.6–108.5)	102.6 (96.2–108.0)	103.2 (97.9–107.7)	99.5 (96.6–106.6)**	96.9 (95.2–107.7)**	94.8 (92.3–106.8)**	95.5 (91.2–105.3)**	94.2 (89.9–104.5)**
Waist-to-hip ratio	1.02 (0.96–1.05)	1.02 (0.96–1.04)	1.01 (0.96–1.05)	1.00 (0.95–1.04)	0.99 (0.94–1.04)**	1.00 (0.94–1.03)**	1.00 (0.93–1.03)**	1.00 (0.92–1.04)**
Total body water (l)	32.7 (28.8–47.8)	32.7 (28.3–46.3)	33.0 (29.0–45.2)	32.9 (28.2–45.0)	33.7 (28.5–44.2)	34.1 (28.2–43.8)	33.1 (28.3–44.3)	33.9 (28.4–43.5)
Body fat mass (kg)	28.3 (25.7–35.4)	29.6 (24.9–34.5)	30.1 (25.9–34.4)	27.3 (24.9–33.0)*	26.0 (23.2–32.9)**	25.0 (21.3–32.5)**	24.7 (19.6–32.3)**	22.2 (18.8–31.4)**
Percent body fat (%)	42.4 (33.0–44.5)	40.0 (33.2–44.0)	41.2 (32.9–43.9)	39.5 (31.5–42.1)	39.9 (30.5–41.8)*	38.2 (30.0–41.3)**	38.6 (30.1–41.7)**	38.2 (27.2–41.0)**
Lean mass (kg)	45.0 (39.2–65.0)	44.3 (38.5–62.7)	44.6 (39.3–61.5)	44.5 (38.4–61.3)	45.6 (38.8–60.0)	46.1 (38.4–59.7)	44.7 (38.5–60.1)	45.8 (38.7–59.2)
Protein (kg)	8.7 (7.7–12.7)	8.6 (7.6–12.3)	8.7 (7.7–12.1)	8.6 (7.6–12.1)	8.7 (7.6–11.9)	8.9 (7.6–11.8)	8.6 (7.5–11.8)	8.8 (7.6–11.7)
Soft lean mass (kg)	41.9 (36.9–61.4)	41.9 (36.3–59.4)	42.2 (37.1–57.9)	42.1 (36.2–57.9)	43.0 (36.6–56.6)	43.6 (36.3–56.3)	42.3 (36.2–56.6)	43.3 (36.5–55.9)
Skeletal muscle mass (kg)	24.6 (21.1–36.3)	24.0 (20.7–34.9)	24.1 (21.1–34.4)	24.1 (20.9–34.6)	24.4 (21.1–33.7)	24.9 (20.9–33.6)	24.0 (20.7–33.6)	24.7 (20.9–33.3)
Percent skeletal muscle (%)	30.9 (30.2–37.2)	32.4 (30.2–37.3)	32.9 (30.4–37.3)	32.4 (31.4–37.3)	33.6 (31.6–37.8)	34.1 (31.7–38.2)*	34.0 (31.3–37.9)*	34.1 (31.9–40.4)**
ASM (%)	23.3 (22.7–28.1)							25.5 (24.3–30.2)**
SMI (kg/m^2^)	7.61 (6.36–9.12)							7.70 (6.19–8.56)

ASM = appendicular skeletal muscle mass/body weight; BMI = body mass index; SMI = skeletal muscle mass index.

* *P* < 0.05 (Wilcoxon signed-rank test).

** *P* < 0.01 (Wilcoxon signed-rank test).

As indicated in Table II, BMI and body weight had decreased significantly by Week 2 of treatment, and both of these continued to decrease during the study, with median values of 27.3 (range, 24.8–31.3) and 75.8 kg (range, 59.8–82.3 kg) at Week 24 (both *P* values < 0.01). The waist-to-hip ratio significantly decreased from 1.02 (range, 0.96–1.05) at baseline to 1.00 (range, 0.92–1.04) at Week 24 (*P* < 0.01). Waist circumference decreased significantly from 101.4 cm (range, 97.6–108.5 cm) at baseline to 94.2 cm (range, 89.9–104.5 cm) at Week 24 (*P* < 0.01) (Table II). Body fat mass decreased significantly from 28.3 kg (range, 25.7–35.4 kg) at baseline to 22.2 kg (range, 18.8–31.4 kg) at Week 24 (*P* < 0.01) (Table II) as did the percentage of body fat (from 42.4% [range, 33.0%–44.5%] to 38.2% [range, 27.2%–41.0%]; *P* < 0.01). The percentage of skeletal muscle mass significantly increased from 30.9% (range, 30.2%–37.2%) at baseline to 34.1% (range, 31.9%–40.4%) at Week 24 (*P* < 0.01) (Table II). There were no significant changes in total body water or lean mass.

Appendicular skeletal muscle (ASM) mass relative to body weight increased significantly from 23.3% (range, 27.7%–28.1%) at baseline to 25.5% (range, 24.3%–30.2%) at Week 24 (*P* < 0.05). There was no change in the skeletal muscle mass index.

### Liver tests

**Figure 2:**
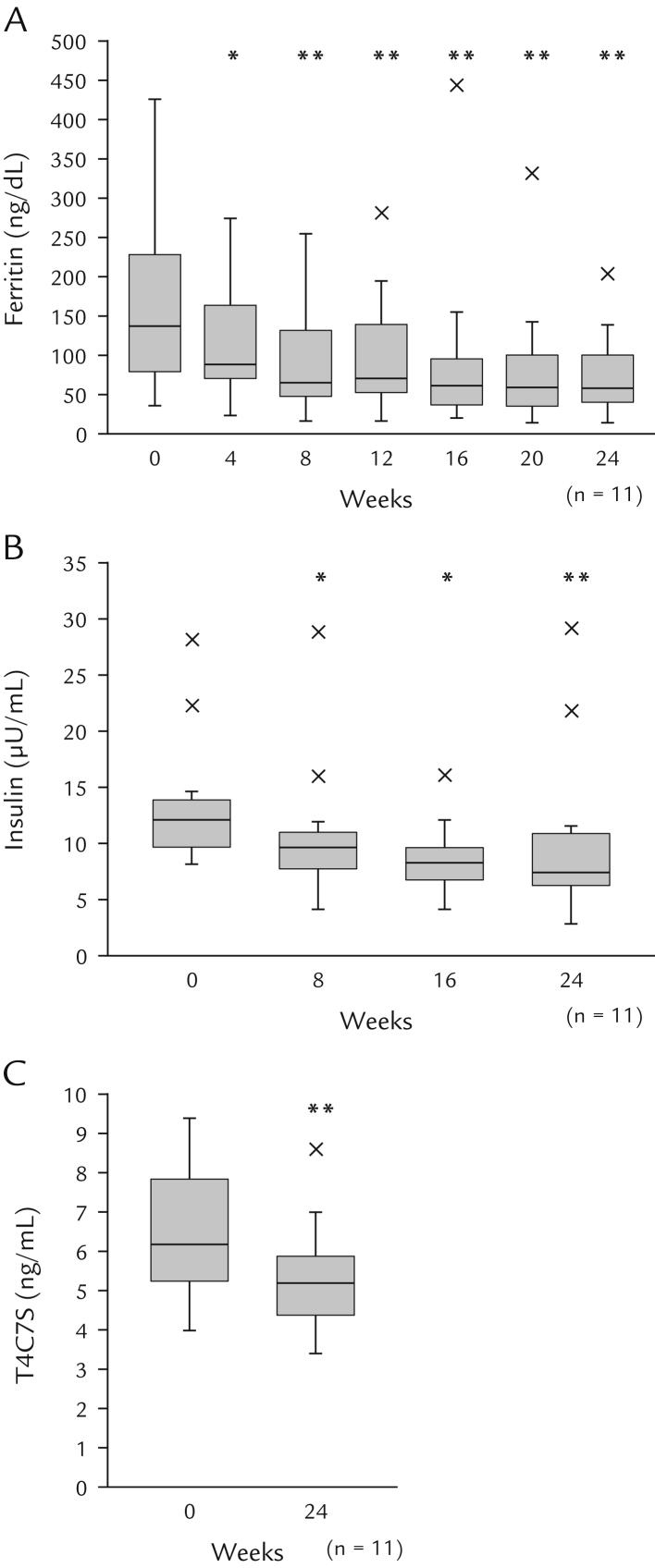
Changes in NAFIC-related variables. Box-and-whisker plots are shown for (A) serum ferritin, (B) insulin, and (C) type IV collagen 7S in 11 patients treated with dapagliflozin for 24 weeks. Data are expressed as the median with interquartile range and maximum values. Outliers are indicated by ×, and were defined as values exceeding the 75th percentile plus 1.5 times the interquartile range. ^*^P < 0.05 and ^**^P < 0.01 versus baseline (Wilcoxon signed-rank test). T4C7S = type IV collagen 7S.

Table III shows the liver tests at baseline and their changes during the study. Serum AST, ALT, and γ-glutamyl transpeptidase concentrations decreased progressively during the study from baseline values of 52 U/L (range, 43–55 U/L), 59 U/L (range, 48–69 U/L), and 64 U/L (range, 47–94 U/L), respectively, to values of 26 U/L (range, 24–38 U/L), 30 (range, 20–37 U/L), and 33 U/L (range, 24–67 U/L), respectively, at Week 24 (all *P* values < 0.01 vs baseline) (Table III). These changes occurred together with significant reductions in serum ferritin, insulin, and T4C7S concentrations (Figure 2). The NAFIC score decreased significantly from 3.0 (range, 1.5–3.0) to 2.0 (range, 0–2.5) (*P* < 0.05). Serum adiponectin concentrations increased significantly from 5.40 µg/mL (range, 4.60–8.85 µg/mL) at baseline to 7.0 µg/mL (range, 5.6–11.8 µg/mL) at Week 24 (*P* < 0.01) (Table III). High sensitivity C-reactive protein concentrations decreased during the study, although not significantly.

**Table III:** Effects of dapagliflozin on liver tests and metabolic laboratory variables (n = 11).

	Week 0 (baseline)	Week 2	Week 4	Week 8	Week 12	Week 16	Week 20	Week 24
	Median (interquartile range)							
AST (U/L)	52 (43–55)	50 (48–60)	48 (41–53)	39 (32–42)**	41 (30–42)**	33 (23–38)**	32 (22–35)**	26 (24–38)**
ALT (U/L)	59 (48–69)	65 (49–76)	55 (47–73)	47 (36–50)**	40 (27–45)**	30 (26–40)**	34 (23–39)**	30 (20–37)**
γ-GTP (U/L)	64 (47–94)	57 (37–86)**	49 (36–81)**	50 (30–69)**	44 (30–69)**	38 (23–65)**	35 (24–67)**	33 (24–67)**
Fibrosis-4 index	1.83 (1.35–2.49)	1.99 (1.13–2.52)	2.01 (1.25–2.90)	1.94 (1.13–2.34)	1.68 (1.06–2.18)*	1.73 (1.12–1.95)**	1.87 (1.06–1.99)	1.59 (1.29–2.37)
Adiponectin (µg/mL)	5.40 (4.60–8.85)		5.90 (4.85–10.15)**		6.60 (4.80–10.15)**			7.00 (5.60–11.80)**
hsCRP (mg/dL)	0.26 (0.11–0.53)		0.14 (0.08–0.26)**	0.13 (0.07–0.40)	0.15 (0.06–0.48)	0.12 (0.09–0.24)	0.20 (0.09–0.29)	0.12 (0.05–0.32)
FPG (mg/dL)	147 (132–176)	138 (121–153)*	126 (121–155)**	124 (115–153)**	128 (114–159)**	120 (106–148)**	117 (109–156)**	119 (107–150)**
HbA1c (%)	7.4 (6.9–8.3)		6.9 (6.5–7.95)**	7.0 (6.25–7.55)**	6.7 (6.15–7.4)**	6.8 (6.15–7.4)**	6.5 (6.0–7.25)**	6.7 (5.95–7.3)**
Glucagon (pg/mL)	184 (166–198)			168 (157–181)*		172 (158–181)		175 (181–198)
HDL-C (mg/dL)	52 (46–58)	52 (46–55)	51 (46–58)	52 (43–58)	49 (45–63)	55 (48–62)*	58 (52–67)**	55 (49–64)*
LDL-C (mg/dL)	116 (106–124)	104 (97–123)	109 (91–127)	118 (94–129)	115 (106–120)	107 (96–119)	119 (108–131)	116 (106–126)
Triglycerides (mg/dL)	118 (111–162)	123 (97–157)	108 (80–145)	99 (90–141)*	96 (86–116)*	97 (79–128)**	95 (80–136)*	98 (84–154)

AST = aspartate aminotransferase; ALT = alanine aminotransferase; FPG = fasting plasma glucose; γ-GTP = γ-glutamyltranspeptidase; HbA1c = glycated hemoglobin; hsCRP = high sensitivity C-reactive protein.

* *P* < 0.05 (Wilcoxon signed-rank test).

** *P* < 0.01 (Wilcoxon signed-rank test).

In terms of the evaluation of hepatic steatosis using abdominal ultrasound, distinct improvement was found in hepatorenal contrast, deep attenuation, or unclear vessels in 8 out of 11 patients, although no clear change was observed in 3 out of 11 patients.

### Metabolic laboratory variables

Table III also shows the baseline values and changes in metabolic laboratory variables. As expected from the mechanism of action of dapagliflozin, FPG decreased from 147 mg/dL (range, 132–176 mg/dL) at baseline to 119 mg/dL (range, 107–150 mg/dL) at Week 24 (*P* < 0.01). Likewise, HbA1c decreased significantly from 7.4% (range, 6.9%–8.3%) to 6.7% (range, 5.95%–7.3%) (*P* < 0.01). Serum glucagon concentrations did not change significantly from baseline to Week 24, although serum HDL-C concentrations significantly increased from 52 mg/dL (range, 46–58 mg/dL) to 55 mg/dL (range, 49–64 mg/dL) (*P* = 0.04), there were no notable changes in LDL-C or triglyceride concentrations.

### Other laboratory variables

As indicated in Table IV, there were no significant changes in general laboratory variables from baseline to Week 24, except for hematocrit.

**Table IV:** General laboratory variables.

	Week 0 (baseline)	Week 2	Week 4	Week 8	Week 12	Week 16	Week 20	Week 24
	Median (interquartile range)							
UA (mg/dL)	6.1 (4.5–6.9)	4.5 (4.2–5.6)*	4.9 (3.9–5.6)	4.7 (3.9–6.2)*	5.1 (3.8–6.0)*	4.3 (4.0–6.2)*	4.8 (3.9–6.4)	5.0 (4.0–6.0)
HT (%)	44.0 (40.9–46.1)	44.0 (42.2–46.4)*	43.2 (41.6–47.6)	45.8 (42.3–47.7)	46.3 (44.0–49.4)**	44.7 (42.9–47.5)*	45.4 (42.8–48.1)**	44.4 (42.3–47.0)**
BUN (mg/dL)	12.5 (11.8–15.8)	12.7 (11.2–17.3)	14.0 (13.6–16.2)	12.9 (11.3–14.9)	15.2 (10.7–16.2)	14.9 (10.9–16.6)	16.2 (13.8–18.0)**	14.7 (13.3–17.1)
Cr (mg/dL)	0.62 (0.48–0.83)	0.64 (0.49–0.83)	0.64 (0.49–0.83)	0.65 (0.50–0.87)	0.68 (0.51–0.87)*	0.65 (0.51–0.79)	0.66 (0.48–0.91)	0.67 (0.47–0.85)
eGFR (mL/min/1.73 m^2^)	92 (79–102)	90 (77–100)	86 (78–102)	86 (76–98)	86 (74–94)	91 (83–100)	83 (71–102)	91 (77–100)
RBC (×10^4^/µL)	483 (432–509)	483 (447–503)	472 (437–518)	506 (457–539)	494 (457–539)**	494 (465–512)**	506 (468–515)**	489 (466–508)
Hb (g/dL)	15.0 (13.7–15.8)	15.4 (14.1–15.9)*	15.2 (13.9–16.2)	15.9 (14.3–16.1)	15.7 (14.5–16.6)*	15.3 (14.5–16.1)	15.3 (14.4–16.2)	15.3 (14.4–16.0)
MCV (fL)	91.9 (90.3–93.6)	91.5 (90.6–94.7)	92.1 (90.7–94.7)	92.5 (90.3–94.7)	92.7 (90.7–94.6)	92.0 (91.0–93.9)	91.8 (89.4–94.5)	92.0 (90.3–93.9)
MCHC (%)	33.5 (33.2–34.2)	33.6 (33.2–34.3)	33.3 (33.1–36.5)	33.4 (33.3–33.8)	33.2 (32.8–33.4)*	33.5 (32.8–33.9)	33.4 (33.1–34.0)	33.4 (32.9–34.3)

BUN = blood urea nitrogen; Cr = creatinine; eGFR = estimated glomerular filtration rate; Hb = hemoglobin; HT = hematocrit; MCHC = mean corpuscular hemoglobin concentration; MCV = mean corpuscular volume; RBC = red blood cell count; UA = urine albumin.

* *P* < 0.05 (Wilcoxon signed-rank test).

** *P* < 0.01 (Wilcoxon signed-rank test).

### Blood pressure

Systolic blood pressure decreased from 126 mm Hg (range, 122–134 mm Hg) at baseline to 120 mm Hg (range, 114–122 mm Hg) at Week 24. Diastolic blood pressure decreased from 84 mm Hg (range, 79–88 mm Hg) at baseline to 70 mm Hg (range, 68–79 mm Hg) at Week 24.

## Discussion

In this study, we showed that the administration of dapagliflozin, a potent and selective SGLT2 inhibitor, was associated with improvements in liver tests and metabolic laboratory variables in patients with NASH and T2DM over the course of 24 weeks. These patients experienced significant reductions in body weight and BMI, which were driven by marked reductions in body fat (total fat mass and percent body fat). Although the percent skeletal muscle mass increased, the actual skeletal muscle mass remained unchanged. Moreover, there were no marked changes in total body water, protein, or soft lean mass during the treatment period.

There were significant decreases in median values of waist circumference and waist-to-hip ratio, which are also suggestive of reduced visceral fat mass. This is important because higher waist circumferences and waist-to-hip ratios caused by visceral fat mass have been strongly associated with an increased risk factor for metabolic and cardiovascular disease in adults.^27^ Because of the deleterious relationship between visceral fat distribution, hepatic insulin resistance, and adiponectin,^28^ we also measured serum adiponectin levels. We found a significant increase in adiponectin, which may be related to the reduction in visceral fat mass in these patients.

The hepatic effects of dapagliflozin were assessed in terms of the changes in liver enzymes, serum ferritin, insulin, and T4C7S. All of these variables showed significant improvements during the 24-week study. We also calculated the NAFIC score and Fibrosis-4 index.^24^, ^25^, ^26^ The NAFIC score can assist in the diagnosis of NASH by using the levels of ferritin, fasting insulin, and T4C7S. The Fibrosis-4 index is a useful parameter when excluding the diagnosis of NASH in patients with advanced fibrosis and takes into consideration ALT, AST, and platelet counts.^29^ We observed a significant reduction in the NAFIC score from baseline to Week 24 owing to the significant reductions in ferritin, insulin, and T4C7S over time. The Fibrosis-4 index was significantly lower at Weeks 12 and 16, but not at Week 24, compared with baseline.

Serum ferritin concentration is an independent predictor of hepatic iron overload, which is associated with NASH and advanced hepatic fibrosis.^29^ Elevation of serum ferritin levels is associated with the severity of fibrosis in NAFLD.^30^ In addition, serum ferritin levels are closely associated with insulin resistance and can be considered a marker for metabolic syndrome.^31^ Although the mechanism underlying the elevation of serum ferritin in NASH is unknown,^32^ the serum ferritin concentration started to decrease in the present study before changes in ALT were observed, which suggests that the reduction in ferritin concentration may have contributed to the improvement in hepatic inflammation. A previous study found that serum ALT levels did not decrease even if the ferritin level in patients with nonalcoholic fatty liver disease was decreased by phlebotomy^33^; therefore, it is necessary to examine whether the decrease in ferritin level actually relates to the decrease in serum ALT level in patients with nonalcoholic steatohepatitis. Hyperinsulinemia and increased insulin resistance could have important roles in the pathogenesis of NASH in both Western and Asian countries.^34^, ^35^, ^36^, ^37^ Hyperinsulinemia in patients with NASH is attributable to increased insulin secretion, which compensates for reduced insulin sensitivity and is not a consequence of decreased hepatic extraction of insulin, which occurs in all forms of chronic liver disease at the stage of advanced fibrosis or cirrhosis.^34^, ^35^ In this study, fasting insulin and fasting plasma glucose decreased together with adiponectin. These results suggest that dapagliflozin improves insulin resistance by decreasing visceral fat mass.

The serum insulin concentrations were significantly lower at Weeks 8, 16, and 24 than at baseline. Insulin resistance is a common factor in NASH and T2DM, and the decrease in insulin secretion coupled with improvements in glycemic control (ie, FPG and HbA1c) likely suggests that there was an improvement in insulin resistance, which might have been partly mediated by the reduction in visceral fat mass and increased adiponectin.

It is also intriguing to note that there were reductions in systolic blood pressure and diastolic blood pressure during the study. The underlying mechanism is unclear; however, it might involve changes in body composition or metabolic variables. It is notable that the changes in blood pressure were not accompanied by significant changes in total body water. The improvement in blood pressure may be particularly relevant to patients with hypertension.

The percentage of skeletal muscle mass increased in this study. A recent Asian study reported that sarcopenia is an independent risk factor for NASH and significant fibrosis.^38^ The authors defined sarcopenia based on the ASM/body weight (ASM%) value. Patients with NASH showed a significantly lower ASM% compared with those without NAFLD. In this study, the ASM% values for NASH patients increased following dapagliflozin treatment. This suggests that dapagliflozin may improve sarcopenia and fibrosis in NASH patients.

A recent retrospective study compared the efficacies of administration of an SGLT2 inhibitor or a DPP4 inhibitor for 24 weeks in Japanese patients with T2DM and biopsy-confirmed NAFLD.^39^ Although serum AST and ALT levels improved significantly in both groups, the reductions in these levels were greater in the SGLT2 inhibitor group than in the DPP4 inhibitor group. The SGLT2 inhibitor group experienced a greater increase in HDL-C, a greater reduction in FPG, and a greater reduction in BMI, but a smaller decrease in the estimated glomerular filtration rate, compared with the DPP4 inhibitor group. A significant reduction in body fat was also observed in the SGLT2 inhibitor group, although body composition was not assessed in the DPP4 inhibitor group. The results of that study are consistent with ours, and indicate that administration of an SGLT2 inhibitor is associated with clinically relevant improvements in liver tests and body composition in patients with NAFLD or NASH.

The safety profile of dapagliflozin was evaluated in terms of general laboratory variables, but there were no clinically significant changes in any of these variables in our cohort during the study. We think that the slight increase in hematocrit was not a result of dehydration, but rather reflective of an increase in red blood cells, because no changes in blood urea nitrogen, creatinine, or estimated glomerular filtration rate were observed. Furthermore, there were no changes in total body water during the study. Thus, dehydration was likely not a complication in the present study. These findings suggest that dapagliflozin did not have any untoward effects on clinically relevant laboratory variables in this small group of selected patients.

The limitations of this study include the open-label design, short treatment period, and the small number of patients. Furthermore, liver biopsies were not performed. Further large-scale, randomized, controlled studies are needed to validate the efficacy of dapagliflozin in the treatment of NASH and T2DM.

In this small, open-label, uncontrolled study, dapagliflozin improved the body composition of patients with NASH associated with T2DM by reducing body fat, most likely visceral fat mass, and was associated with improvements in liver tests and metabolism. Longer-term, larger studies are needed to verify these results and enable the use of dapagliflozin to treat NASH associated with T2DM.

## Conflicts of Interest

Dapagliflozin was developed by AstraZeneca, who provided funding for publication fees and development of the manuscript, but did not play any other role in the study design; collection, analysis, and interpretation of data; writing of the manuscript; or the decision to submit the manuscript for publication. HT and YK have received honoraria from AstraZeneca K.K. Funding to pay for editorial support and the Open Access publication charges for this article were provided by AstraZeneca K.K. and Ono Pharmaceutical Co, Ltd. The authors have indicated that they have no other conflicts of interest regarding the content of this article.
